# Copy number profiling of circulating free DNA predicts transarterial chemoembolization response in advanced hepatocellular carcinoma

**DOI:** 10.1002/1878-0261.13170

**Published:** 2022-01-10

**Authors:** Xiuqing Dong, Geng Chen, Xinghui Huang, Zhenli Li, Fang Peng, Hengkai Chen, Yang Zhou, Lei He, Liman Qiu, Zhixiong Cai, Jingfeng Liu, Xiaolong Liu

**Affiliations:** ^1^ College of Chemical Engineering Fuzhou University China; ^2^ The United Innovation of Mengchao Hepatobiliary Technology Key Laboratory of Fujian Province Mengchao Hepatobiliary Hospital of Fujian Medical University Fuzhou China; ^3^ The Liver Center of Fujian Province Fujian Medical University Fuzhou China; ^4^ Department of Interventional Radiology Mengchao Hepatobiliary Hospital of Fujian Medical University Fuzhou China; ^5^ Liver Disease Center The First Affiliated Hospital of Fujian Medical University Fuzhou China; ^6^ The Hepatobiliary Medical Center of Fujian Province Fujian Cancer Hospital & Fujian Medical University Cancer Hospital Fuzhou China

**Keywords:** circulating free DNA, copy number variants, hepatocellular carcinoma, transarterial chemoembolization, tumour fraction

## Abstract

Transarterial chemoembolization (TACE) is the most commonly used treatment for advanced hepatocellular carcinoma (HCC), but still lacks accurate real‐time biomarkers for monitoring its therapeutic efficacy. Here, we explored whether copy number profiling of circulating free DNA (cfDNA) could be utilized to predict responses and prognosis in HCC patients with TACE treatment. In total, 266 plasma cfDNA samples were collected from 64 HCC patients, 57 liver cirrhosis (LC) patients and 32 healthy volunteers. We performed low‐depth whole‐genome sequencing (LD‐WGS) on cfDNA samples to conduct copy number variant (CNV) analysis and tumour fraction (TFx) quantification. Then, the correlation between TFx/CNVs and therapeutic efficacy, treatment outcomes and lipiodol deposition were explored. The change in TFx during TACE treatment was associated with patients' tumour burden, and could accurately and earlier predict treatment response and prognosis, providing an alternative strategy other than mRECIST. Meanwhile, the chromosomal 16q/NQO1 amplification indicated worse therapeutic response; in patients who underwent multiple TACE sessions, TFx change during their first TACE treatment reflected the long‐term survival; additionally, the copy number amplification of chromosome 1q, 3p, 6p, 8q, 10p, 12q, 18p or 18q affected lipiodol deposition. Overall, we have provided a new liquid biopsy approach for future TACE management of HCC patients.

## Graphical abstract

Accurate evaluation of transarterial chemoembolization (TACE) therapeutic efficacy would greatly benefit hepatocellular carcinoma (HCC) management. In this study, we investigated the copy number profiles of cfDNA and their clinical utilization for predicting TACE responses using low‐depth whole‐genome sequencing. The results demonstrated that cfDNA copy number profiling could accurately predict treatment response and prognosis, thus providing an alternative strategy for guiding TACE management in patients with HCC.

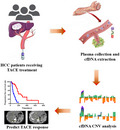

**Abbreviations**
AFP: alpha‐fetoprotein
cfDNA: circulating free DNA
CNVs: copy number variants
CT: computed tomography
HCC: hepatocellular carcinoma
LD‐WGS: low‐depth whole‐genome sequencing
MRI: magnetic resonance imaging
OS: overall survival
PD: progressive disease
PFS: progression‐free survival
PR: partial response
PVTT: portal vein tumour thrombus
SD: stable disease
TACE: transarterial chemoembolization
TFx: tumour fraction

## 1 Introduction

Hepatocellular carcinoma (HCC) is one of the most common malignancies in China, ranking as the 6th highest in incidence and 3rd highest in mortality worldwide [1, 2]. Currently, a considerable portion of HCC patients were diagnosed at advanced stages, leading to low surgical resection rates and poor prognosis. Transarterial chemoembolization (TACE), which could interrupt tumour's blood supply by injecting small embolic particles through a catheter into the hepatic artery and further induce tumour necrosis and apoptosis, is the major first‐line treatment for advanced HCC, and the patients usually receive several TACE cycles in every 4–8 weeks if required. In clinical, in‐time evaluation of TACE response in advanced HCC patients is crucial for guiding subsequent treatment decisions. mRECIST assessment is recommended for evaluating the efficacy of TACE treatment based on reduction of the arterially hyperenhancing viable tumour diameter by computed tomography (CT) or magnetic resonance imaging (MRI). However, image assessment‐like mRECIST is easily interfered by numerous factors (e.g. tumour location, lipiodol deposition, coagulative haemorrhage, necrosis) and greatly relies on the experiences of radiologists, which make it relatively subjective and induce difficulties in accurately evaluation of TACE therapeutic efficacy. Meanwhile, image assessment is also less time‐sensitive. Therefore, there is an urgent need to develop an alternative early evaluation strategy for monitoring TACE therapeutic efficacy, which could prevent patients from undergoing an ineffective therapy and help quickly adapting into other treatment strategies with more clinical benefits.

Circulating free DNA (cfDNA) is short fragmented DNA in the peripheral blood derived from necrotic or apoptotic cells, which could carry tumour‐specific genetic alterations, including single‐nucleotide variants (SNVs), copy number variants (CNVs) and epigenetic variants in cancer patients [3]. Based on these characteristics, cfDNA could be utilized for early diagnosis, efficacy monitoring and prognosis evaluation in HCC [4, 5, 6, 7]. Our previous study has shown that SNVs and CNVs identified from cfDNA, both are highly consistent with the genetic profiles of HCC tissues, could well real‐time reflect tumour burden [8, 9]. Furthermore, when compared with SNVs, CNVs detection in cfDNA only required low‐depth whole‐genome sequencing (LD‐WGS, as low as 0.1× coverage) and thus has the advantages of short detection time and low cost, making it more suitable for rapid diagnosis and efficacy monitoring. Indeed, recent reports have also highlighted the utilization of tumour‐specific CNVs for tumour burden evaluation, which provided a solid strategy for monitoring tumour progression and assessing therapeutic responses without requirement of prior knowledge of tumour genetic information [10, 11]. However, whether cfDNA copy number assessment could real‐time reflect therapeutic responses and prognosis of advanced HCC patients with TACE treatment and which kinds of copy number alteration in HCC patients might be suitable for evaluating TACE treatment were still largely unclear.

In this study, we deployed a well‐applicated algorithm, namely ichorCNA, to assess the copy number profiles of cfDNA and quantify tumour fraction (TFx) in HCC patients receiving TACE treatment. For algorithms such as ichorCNA, a specific panel of normals (PON) that were processed and sequenced similarly to the included cfDNA samples could remove systematic biases of the experimental processes and thus reduce noise and improve accuracy. Thus, to optimize the performance of the ichorCNA algorithm, we created a population‐specific panel of normals, which is constructed based on a reference dataset contained LD‐WGS data of cfDNA from 32 healthy volunteers and 57 liver cirrhosis (LC) patients. The CNVs and quantified TFx of cfDNA were then assessed by optimized ichorCNA in 64 advanced HCC patients who underwent TACE treatment to evaluate their performance in response monitoring and prognosis prediction. Finally, the correlations between cfDNA CNVs and lipiodol deposition were also explored. Overall, our results assessed the CNV profiles of HCC patients receiving TACE treatments for the first time, explored the TFx’s dynamic change during TACE and investigated how these genetic features might affect therapeutic response, providing novel strategies for assessing treatment responses and prognosis of TACE in advanced HCC patients.

## 2 Materials and methods

### 2.1 Patients and sample collection

In total, 64 advanced HCC patients, 57 LC patients and 32 healthy volunteers were enrolled in this study. All HCC patients received TACE treatment at Mengchao Hepatobiliary Hospital of Fujian Medical University after diagnosed with advanced HCC. Clinicopathological and demographic data for each HCC patient were collected from Hospital Information System (HIS). All human sample collection and usage were in accordance with the principles of the Declaration of Helsinki and approved by the Ethics Committee of Mengchao Hepatobiliary Hospital of Fujian Medical University. The written informed consents were also obtained from all the participated individuals.

For HCC patients receiving TACE treatment, blood samples were collected at pretreatment, post‐treatment and long‐term follow‐up time points (from 20 to 314 days). Plasma was separated using two‐step process of centrifugal separation: First, blood samples were centrifuged at 800 ***g*** for 20 min within 2 h of collection, and then, the supernatants were transferred into new tubes and centrifuged again at 17 000 ***g*** for 10 min to remove cell debris. The plasma was then stored at −80 °C until use.

### 2.2 cfDNA extraction and LD‐WGS

cfDNA was extracted from plasma using the QIAamp Circulating Nucleic Acid Kit (Qiagen, Valencia, CA, USA) according to the manufacturer’s instructions. The concentration and quality of cfDNA samples were assessed using Qubit2.0 (Thermo Scientific, Waltham, MA, USA) and Qsep100 (BiOptic, Taipei, China). Library construction of cfDNA was performed using the QIAseq™ cfDNA All‐in‐One Kit, and then, LD‐WGS was conducted on Illumina Nova‐seq 6000 sequencer at Fulgent (Fuzhou, Fujian, China) Technologies Co., Ltd, achieving an average of 3× coverage.

### 2.3 mRECIST evaluation and lipiodol deposition rate calculation

mRECIST was assessed by at least two experienced radiologists based on the postoperative MRI/CT images within 1–2 months, and HCC patients were classified into four categories, namely complete response (CR): disappearance of all target lesions; partial response (PR): at least a 30% decrease in the sum of diameters of viable target lesions; progressive disease (PD): minimum 20% increase in the sum of the diameters of viable target lesions; and stable disease (SD): between PR and PD. Lipiodol deposition rate was calculated also based on CT images within 1–2 months of treatment according to the area of lipiodol and the area of the whole tumour.

### 2.4 Measurement of serum protein biomarker

Commercial Lumipulse G AFP‐N Kit (Fujirebio, Tokyo, Japan) was used to detect the concentration of alpha‐fetoprotein (AFP) in HCC patients’ serum according to the corresponding manufacturer’s instructions. Threshold for AFP abnormality was defined as 20 ng·mL^−1^.

### 2.5 Copy number analysis

Low‐depth whole‐genome sequencing data were mapped and aligned to the hg19 reference using Sentieon version 201911.01) with default parameters. Copy number variation and TFx of plasma samples were calculated using ichorCNA [10], which implemented a hidden Markov model to predict large‐scale copy number alterations for estimating the TFxs based on obtained copy number information. To make this algorithm better fitting for our dataset, a customized population‐specific panel of normals was firstly generated using all plasma samples from healthy volunteer and liver cirrhosis patients, with segment window length set as 10 00 000. Then, the ichorCNA was deployed with default parameters to quantify the TFx for all included plasma samples with abovementioned panel of normals. After the first run, samples with TFx < 5% were extracted, and ichorCNA was redeployed with parameters more suitable for evaluating low TFx for these samples. Detailed parameters are set as follows: initialize TFx parameter: 0.95, 0.99, 0.995, 0.999; initial ploidy: 2; maxCN: 3; estimate ScPrevalence: False; and analysis chromosomes: chr1‐chr22. The final results were then integrated for downstream analysis.

To summary and visualize the CNV distribution of all included cfDNA samples, we deployed CNViewer. First, the samples were categorized according to TFx change or lipiodol deposition rate. Then, the ichorCNA results for each group of samples were extracted and merged. Last, CNViewer was utilized to provide visualization, with figure background all set to blank for clearer demonstration.

### 2.6 Statistical analysis

Statistical analysis was performed using r (version 4.0.3, R Foundation for Statistical, Vienna, Austria). The sensitivity and specificity of ichorCNA model were analysed by receiver operating characteristic (ROC) curves. The correlation of TFx and clinicopathological features was evaluated by chi‐squared tests in cross tables. The potential correlations between TFx and tumour size were analysed using Pearson linear correlation analysis. Kaplan–Meier analysis with log‐rank test was conducted to compare progression‐free survival (PFS) and overall survival (OS) among different groups of patients. Univariate cox proportional hazards regression analysis was performed to evaluate the prognostic value of TFx change and patients' clinical characteristics. Associations were expressed as hazard ratios (HR) with 95% confidence intervals (CI). Student's *t*‐test was used to compare the CNV alteration levels between the two lipiodol deposition groups. *P* < 0.05 was considered statistically significant for all statistical analyses.

## 3 Results

### 3.1 Study design and the patient cohort

**Fig. 1:**
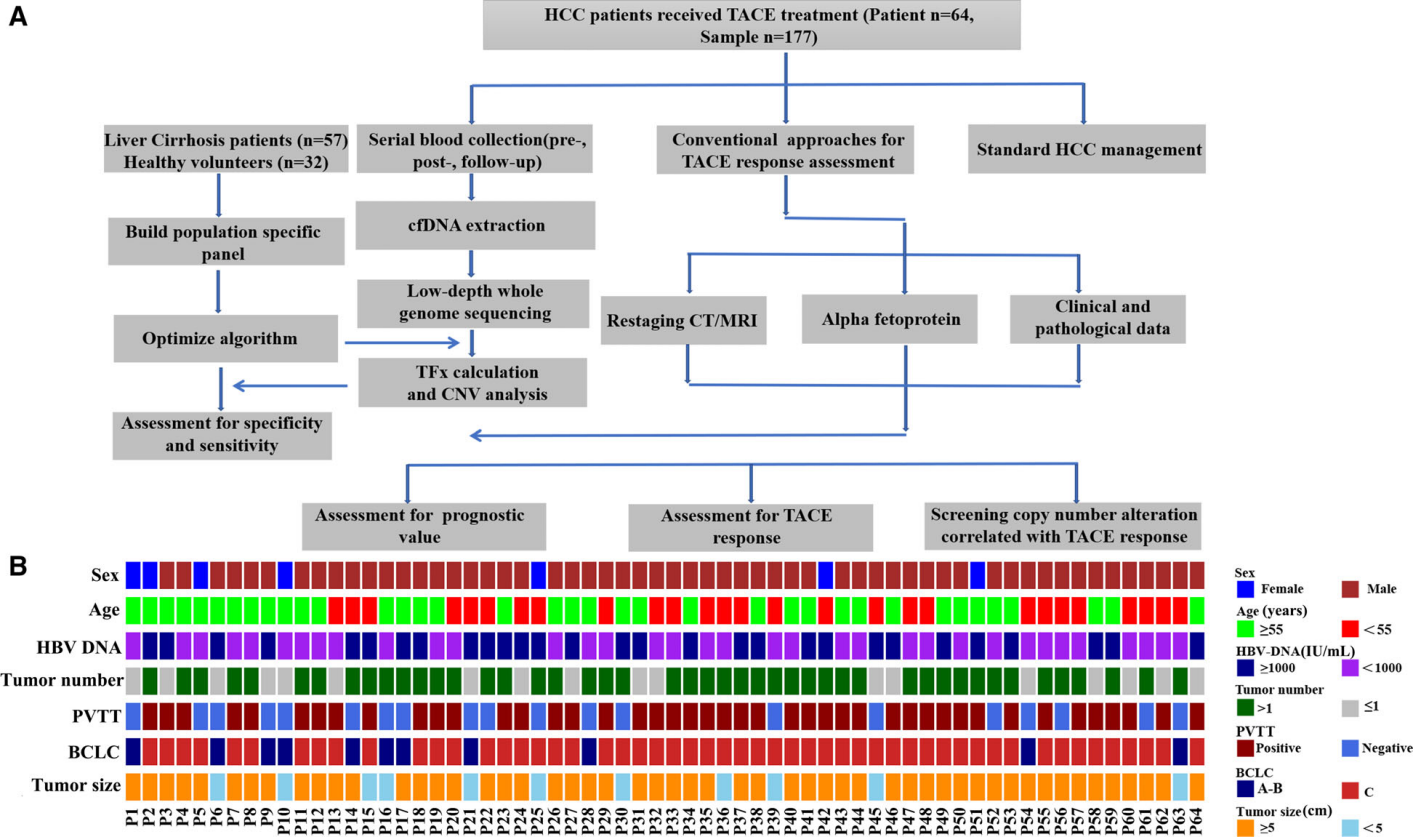
Overview of the study design, patient enrollment and clinical data collection. (A) The flow chart of study design. A total of 64 HCC patients receiving TACE treatment, 57 cirrhosis patients, and 32 healthy volunteers were enrolled. (B) Clinical features of enrolled 64 advanced HCC patients. The clinical and pathological data are coloured according to their categorization.

To investigate the dynamics of cfDNA CNV profiles in advanced HCC patients during TACE treatment, a total of 153 participants, including 32 healthy volunteers, 57 LC patients and 64 advanced HCC patients, were enrolled. The detailed study design was shown in Fig. 1A.

In total, 266 plasma samples were collected. Among these plasma samples, 32 and 57 were collected from healthy volunteers and LC patients, respectively, which serve as reference for downstream analysis; 64 samples were collected from advanced HCC patients before their first TACE treatments, and another 64 samples were collected from them within 1–2 months after TACE treatment; additional 49 samples were collected from 24 long‐term follow‐up HCC patients, of which 16 patients received 2 TACE sessions, 6 patients received 3 TACE sessions, 1 patient received 4 TACE sessions, and 1 patient received 5 TACE sessions.

The clinical characteristics before TACE treatment for enrolled HCC patients were presented in Fig. 1B. In these enrolled HCC patients, the median diameter of tumours was 9.95 cm (range, 1.22–18.8 cm), and 71.8% of them had multiple tumours. Portal vein tumour thrombus (PVTT) was seen in 68.8% (44/64) HCC cases and 82.8% (53/64) of them were classified as Barcelona Clinic Liver Cancer Stage (BCLC) C.

### 3.2 The profiling of cfDNA TFx and CNVs in advanced HCC patients during TACE treatment

cfDNA from all enrolled plasma samples was subject to LD‐WGS with a mean depth of 3.1× (range, 2.35–5.08). Then, CNVs and TFx were analysed using ichorCNA. To evaluate the performance of TFx, we first conduct a benchmark testing to calculate TFx’s power to discriminate HCC patients from healthy volunteers/LC patients. The mean values of TFx in HCC patients, healthy volunteers and LC patients were 0.142 (range: 0–0.653), 0.008 (range: 0–0.118) and 0.006 (range: 0–0.105) respectively (Fig. S1A). The TFx in HCC patients is significantly higher than both healthy volunteers and LC patients, while no significant difference was observed between volunteers and LC patients. ROC curve also revealed that TFx demonstrated excellent performance, achieving a sensitivity of 85.3% and a specificity of 94.4% at cut‐off of 0.016245 (Fig. S1B). This result indicated that the TFx obtained from ichorCNA could provide an accurate tool for estimating tumour burden of HCC patients.

**Fig. 2:**
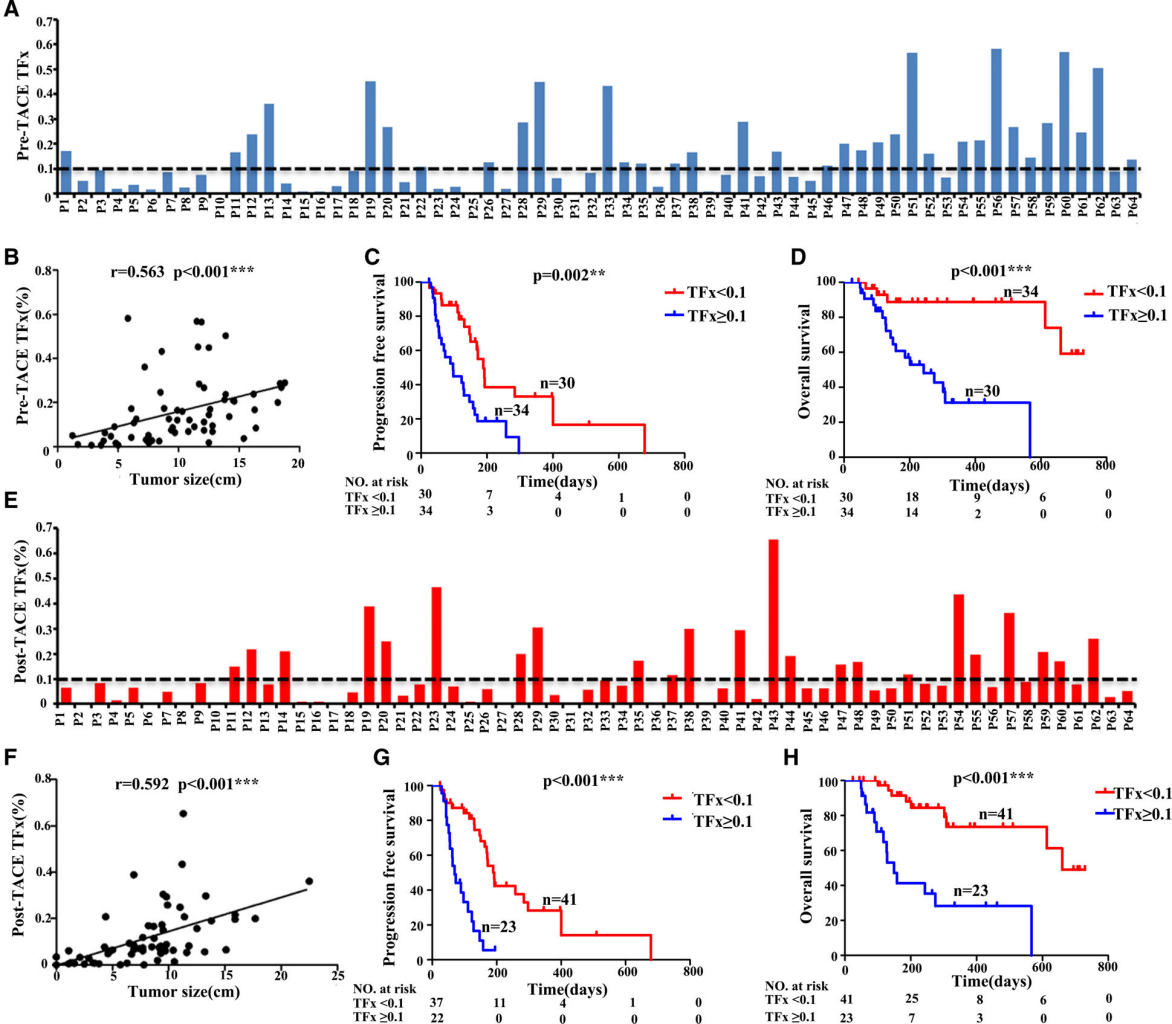
The correlation between TFx and patients' prognosis. (A) The distribution of TFx before TACE (pre‐TACE TFx) in each sample. (B) Pearson correlation and linear regression analysis to show the correlation between pre‐TACE TFx and matched tumour size. (C, D) Kaplan–Meier curves showing PFS(C) and OS(D) for enrolled patients stratified by pre‐TACE TFx, respectively. (E) The distribution of TFx after TACE (post‐TACE TFx) in each sample. (F) Pearson correlation and linear regression analysis to show the correlation between post‐TACE TFx and matched tumour size. (G, H) Kaplan–Meier curves showing PFS(G) and OS(H) for enrolled patients stratified by post‐TACE TFx, respectively. Statistical analysis for Kaplan–Meier plots used the log‐rank test. TFx, tumour fraction; TACE, transarterial chemoembolization; PFS, progression‐free survival; OS, overall survival.

In enrolled HCC patients, 58 of 64 patients (90.6%) showed detectable level of CNVs and TFx in cfDNA before initiating TACE treatment, with mean TFx value of 0.159 (range: 0.006–0.581), while only 78.1% of them showed positive level of AFP (> 20 ng·mL^−1^; Fig. S1C). The distribution of TFx across pre‐TACE samples from all enrolled patients was shown in Fig. 2A. Meanwhile, cfDNA CNVs profiling showed that the top 10 most frequent CNVs in enrolled HCC patients were copy number gain on the regions of chromosomes 1q, 6p, 8q, 20q, 20q and copy number loss on the regions of chromosomes 4q, 13q, 8p, 16q and 17p (Fig. S1D), which were well consistent with the CNV profiling results of HCC tissue samples [12, 13, 14].

Pre‐TACE TFx reflected patients' tumour burden before TACE treatment. As we expected, pre‐TACE TFx showed significant linear correlation with tumour size (*r* = 0.563, *P* < 0.001; Fig. 2B). To further investigate whether pre‐TACE TFx could provide prognostic relevant classification of patients, we divided HCC patients into two groups using TFx of 0.1 as the cut‐off value (Fig. 2A), which is considered as prognostic threshold in several previous studies [10, 15]. The Kaplan–Meier analysis showed that the patients with high TFx had shorter PFS (median survival 97 versus 189 days, *P* = 0.002) and OS (median survival 243 versus 630 days, *P* < 0.001; Fig. 2C,D). Meanwhile, chi‐square analysis also confirmed that the presence of PVTT and the level of HBV‐DNA were significantly positively associated with TFx (Table S1). We further performed multivariable logistic analysis using these significant factors, and only HBV‐DNA remained as the significant independent factor related to pre‐TACE TFx (Table S2). Taken together, these results suggested that pre‐TACE TFx could serve as an effective prognostic biomarker reflecting tumour burden in HCC, providing classification for better clinical evaluation.

Since post‐TACE TFx represented patients' tumour burden after treatment, we also analysed post‐TACE TFx within 1‐2 months after TACE for enrolled HCC patients. As shown in Fig. 2E, 81.3% (52/64) of HCC patients still showed a detectable level of TFx in post‐TACE plasma samples and 35.9% (23/64) showed TFx ≥ 0.1, while abnormal level of AFP was only showed in 70.3% patients (Fig. S1E). As expected, post‐TACE TFx was also significantly associated with tumour size (*r* = 0.592; *P* < 0.001; Fig. 2F). Dividing patients according to post‐TACE TFx (cut‐off value: 0.1) also revealed that the patients with high post‐TACE TFx showed significantly shorter PFS (median survival 69 versus 192 days, *P* < 0.001) and OS (median survival 149 versus 660 days, *P* < 0.001). Meanwhile, chi‐square analysis also confirmed that the presence of PVTT, tumour number and tumour size was significantly positively associated with post‐TACE TFx (Table S3). Then, we further performed multivariable logistic analysis using these significant factors, and only the tumour size remained as the significant independent factor related to post‐TACE TFx (Table S4). Compared with pre‐TACE TFx, post‐TACE TFx showed more significant association with patients' prognosis and thus might serve as a better prognostic indicator for patients’ survival (Fig. 2G,H).

### 3.3 Therapeutic efficacy assessment of TACE treatment using TFx

**Fig. 3:**
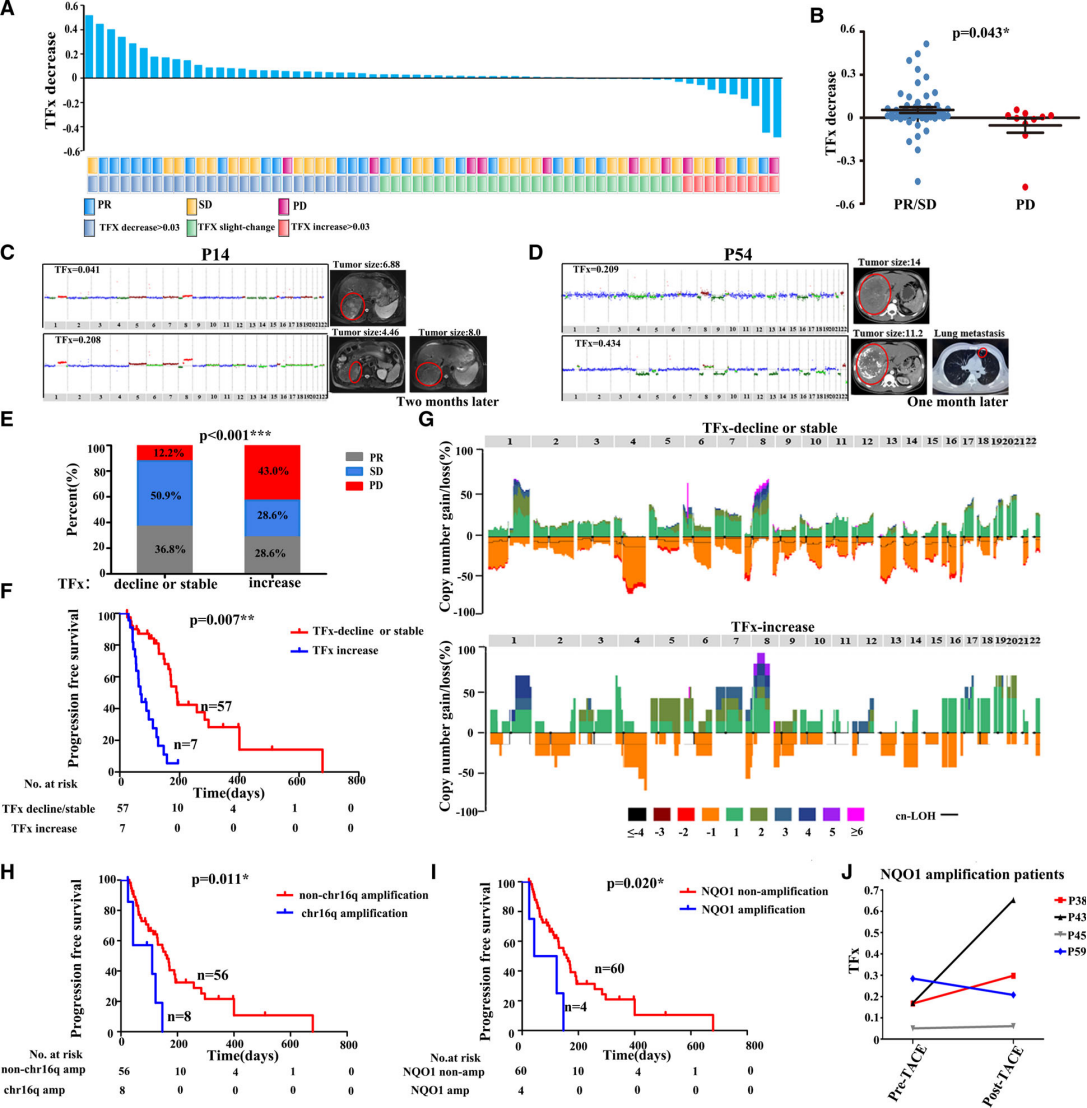
Therapeutic efficacy assessment of TACE based on TFx in HCC patients. (A) Agreement between TFx variations and mRECIST criteria. The waterfall plot shows the change between pre‐TACE TFx and post‐TACE TFx. (B) TFx change in the PR/SD and PD group. The error bars indicate SEM. (C, D) Comparison of TFx with corresponding CT/MRI imaging during the TACE treatment. Red circle indicates the tumour location. Copy number amplification (red), copy number neutral (blue) and copy number deletion (green) are indicated. (E) Proportional representation of PR, SD, PD in TFx decline/stable and TFx increase groups. (F) Kaplan–Meier curves showing PFS in patients with TFx decline/stable versus TFx increase. (G) Copy number profile of the TFx decline/stable and TFx increase groups. Patterns above the x‐axes represent amplification, whereas opposite contours indicate deletion and horizontal black lines indicate loss of heterozygosity. (H, I) Kaplan–Meier curves showing PFS in patients with amplification versus nonamplification in the long arm of chromosome 16 and NQO1, respectively. (J) TFx change between pre‐TACE TFx and post‐TACE TFx in 4 patients with NQO1 amplification. Student *t*‐test was used to compare TFx change between 2 groups of PR/SD and PD. Chi‐squared test was used to compare proportional representation of PR, SD, PD between TFx decline/stable and TFx increase groups. Statistical analysis for Kaplan–Meier plots used the log‐rank test. TFx, tumour fraction; TACE, transarterial chemoembolization; PR, partial response; PD, progressive disease; SD, stable disease; CT, computed tomography; MRI, magnetic resonance imaging.

It is well known that mRECIST based on image diagnosis was the gold standard for efficacy assessment of TACE treatment, but was susceptible to the interference of certain factors such as the experience of radiologists. According to the mRECIST criteria, among all enrolled 64 HCC patients receiving TACE treatment, 10 patients were considered as progression of disease (PD); 31 patients were with SD; and 23 patients showed PR. To investigate whether the dynamic change in TFx could reflected the therapeutic efficacy, we calculated the TFx decrease between pre‐ and post‐TACE groups, which is shown in Fig. 3A. The threshold for absolute value change was set to 0.03, which is commonly considered as the sensitivity of ichorCNA using LD‐WGS in detecting tumour burden. In 42.2% of patients, we observed decrease in post‐TACE TFx > 0.03, 14.1% patients showed increase in TFx > 0.03, while other 43.7% patients only showed almost no change of TFx. Furthermore, the TFx change after TACE showed significant difference between patients achieving PR/SD and PD (median decrease of 0.0540 versus median increase of 0.0539, *P* = 0.043; Fig. 3B), suggesting that the TFx change was in well agreement with mRECIST’s assessment. Moreover, combining a detailed analysis of patient’s whole clinical course, we discovered that monitoring TFx also provided a noninvasive approach to predict tumour relapse ahead of imaging diagnosis. Take HCC patient P14 as the example, who had been evaluated as PR by mRECIST, showed increased TFx after TACE treatment, suggesting that TACE did not successfully reduce his tumour burden, which is confirmed by the fact that CT/MRI imaging reported significant enlargement of tumour 2 months later (Fig. 3C). Similarly, HCC patient 54 (P54) showed SD after TACE, while the increase TFx hinted that tumour burden did not get suppressed by TACE treatment. Indeed, 1 month later, CT/MRI imaging reported a metastasis lesion in the lung (0.5 × 0.3 cm), showing that TFx could serve as an early marker for tumour relapse, which are even more time‐sensitive than traditional imaging strategies (Fig. 3D). Taken all together, the TFx might provide a supplementary to traditional imaging‐based strategies.

Based on above results, we further explored the prognostic value of TFx change in HCC. First, we grouped enrolled patients according to the fold change and absolute value change in TFx (the threshold for relative change was set to 50% and the threshold for absolute value change was set to 0.03), including TFx decline: relative decrease in TFx was greater than 50% and the absolute value of TFx change was > 0.03; TFx increase: increase in TFx was > 50% and the absolute value of TFx change was > 0.03; TFx stable: no abovementioned dramatic change in TFx was observed. Among 64 enrolled patients, 14 patients were grouped as TFx decline; 43 were TFx stable; and 7 were TFx increase. As shown in Fig. 3E, the PR and SD groups in the mRECIST criteria were significantly overlapped with TFx decline and TFx stable groups, and the PD group was significantly enriched in TFx increase group. As expected, TFx change classification showed a striking correlation with PFS, where patients with TFx decline or TFx stable had a median survival of 163 days, comparing to just 63 days in patients with TFx increase (*P* = 0.007; Fig. 3F). Meanwhile, the Cox regression analysis also showed that TFx change classification could further serve as an indicator for PFS (*P* = 0.011; Table S5).

Genetic profiles of tumour have been reported to affect patients' response to treatment. To determine whether copy number change in certain genomic region in cfDNA has impact on therapeutic efficacy, we summarized all gains and losses of copy numbers in cfDNA before patients received TACE and compared their differences between patients categorized as TFx increase and patients categorized as either TFx decline or TFx stable. As shown in Table S6 and Fig. 3G, only chromosome 16p gain was significantly enriched in TFx increase group (*P* = 0.049), suggesting that chromosomal amplification of 16p is associated with poor therapeutic efficacy of TACE in HCC patients. Indeed, Kaplan–Meier curves for PFS confirmed that patients with chromosome 16p amplification in cfDNA before TACE had a significantly shorter PFS than those without amplification (median survival 111 days versus 163 days, *P* = 0.011; Fig. 3H). To further understand which gene might play crucial parts in this association, we extracted driver genes on 16p according to DriverDB(v3), which included 22 genes. Among these genes, 4 of them showed expression significantly associated with the prognosis of HCC patients, including NQO1, GPT2, ZDHHC7 and DHODH. Noteworthily, NQO1 has been reported with capability to induce drug resistance against chemotherapeutic agents [16, 17], which make it a fit candidate for our downstream analysis. In all enrolled HCC patients, 4 of them showed NQO1 amplification in cfDNA. As we expected, the efficacy of TACE treatment in these 4 patients was relatively poor, and their PFS was significantly shorter than HCC patients without NQO1 amplification (median survival 83 versus 158 days, *P* = 0.020; Fig. 3I,J). These results suggest that the copy number variations of NQO1 might serve as a potential marker for efficiency of TACE treatment, which could help determine whether patients could benefit from TACE treatment.

In summary, our analysis revealed that TFx derived from liquid biopsy provided a powerful tool to monitor patients' response to TACE treatment, which could become a potential supplement to traditional imaging approach, with its dynamics directly reflecting patients’ clinical response. The genetic profiling of cfDNA also could be utilized to better optimize clinical decisions, presenting an exciting opportunity for guiding personized TACE treatment.

### 3.4 Comprehensive TFx profiles in long‐term follow‐up patients with TACE treatment

**Fig. 4:**
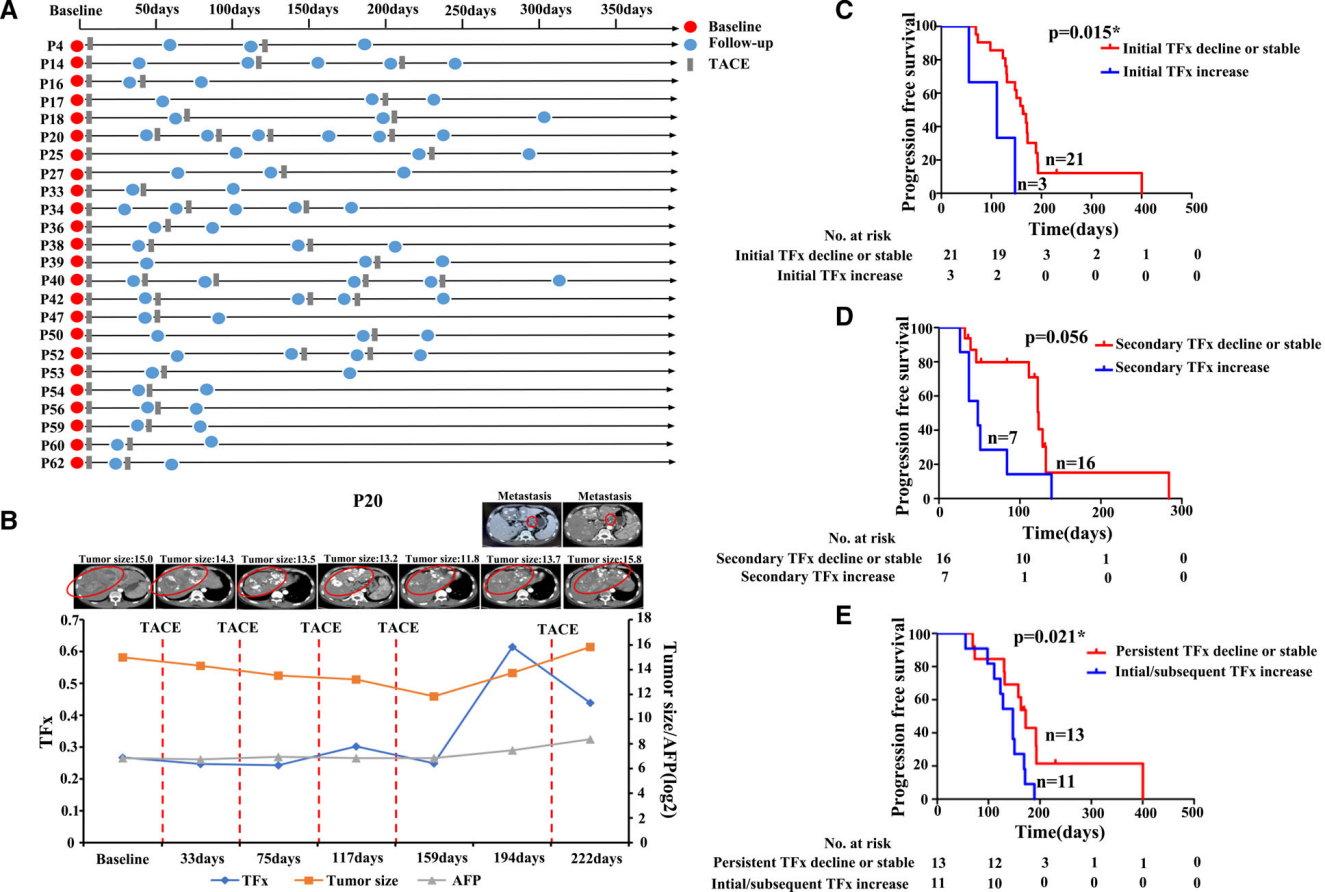
Comprehensive profiles of TFx in long‐term follow‐up patients. (A) The timeline presentation of follow‐up cfDNA samples in 24 HCC patients who received two or more TACE sessions. (B) The time‐course demonstration of TFx score (blue line), serum biomarker AFP level (orange line), tumour size (grey line) and corresponding CT/MRI imaging in patient 20. Red circle indicates the tumour location. (C, D) Kaplan–Meier curves showing PFS for long‐term follow‐up patients stratified by initial TFx change(C), secondary TFx change(D), respectively. (E) Kaplan–Meier curves showing PFS in patients with persistent TFx decline or stable versus initial and subsequent TFx increase. Statistical analysis for Kaplan–Meier plots used the log‐rank test. TFx: tumour fraction; AFP, alpha‐fetoprotein; CT, computed tomography; MRI, magnetic resonance imaging; PFS, progression‐free survival.

Clinically, some HCC patients require repeated TACE therapy to achieve the best antitumour response, which provided valuable resource to further explore how TFx might dynamically track tumour burden during long‐term clinical courses. By retrospectively analysing 24 HCC patients who have received more than one TACE sessions (range: 2–5; Fig. 4A), we discovered that TFx of cfDNA dynamically correlated with patients’ tumour burden during the whole clinical course, showing high consistence with imaging results. For example, as shown in Fig. 4B, Patient 20 (P20) received five TACE sessions during his clinical course and the blood samples were collected at 7 time points. CT imaging results showed that his tumour remained as a SD status during the first four TACE sessions and become PD with metastasis at 194 days before the last TACE session, which still remain visible after TACE at 222 days. This observation is well consistent with the dynamical change of TFx in cfDNA, which remained relatively stable during the first 4 TACE sessions and showed notable increase (from 0.249 to 0.645) at the Day 194. Similar results were also observed in other patients (Fig. S2). In Patients P14 and P54, the TFx change could even predict tumour progression before visible features could be monitored by CT/MRI scans. Taken together, these results suggest that monitoring TFx in HCC patients' longitudinal plasma samples during TACE treatment may provide an accurate and real‐time strategy for tumour burden evaluation.

It has been long debated whether patients’ response to the first TACE session could predict the outcome of whole clinical course [18, 19, 20]. Comparison of patients' PFS revealed that patients showed nonincreasing of TFx after first TACE session could achieve significantly better prognosis (median survival 111 versus 163 days, *P* = 0.015; Fig. 4C), while such phenomenon was not observed for TFx change during second TACE session, suggesting that the first session of TACE has significant impact on patients' disease progression (Fig. 4D). Furthermore, when classify patients into persistent TFx decline or stable group and initial/subsequent TFx increase group, we found that persistent TFx decline or stable group had better PFS (median survival 147 versus 172 days, *P* = 0.021; Fig. 4E), suggesting that TFx could serve as a long‐term biomarker for monitoring patients’ disease progression.

### 3.5 Correlation between lipiodol deposition and CNVs in pre‐TACE cfDNA

**Fig. 5:**
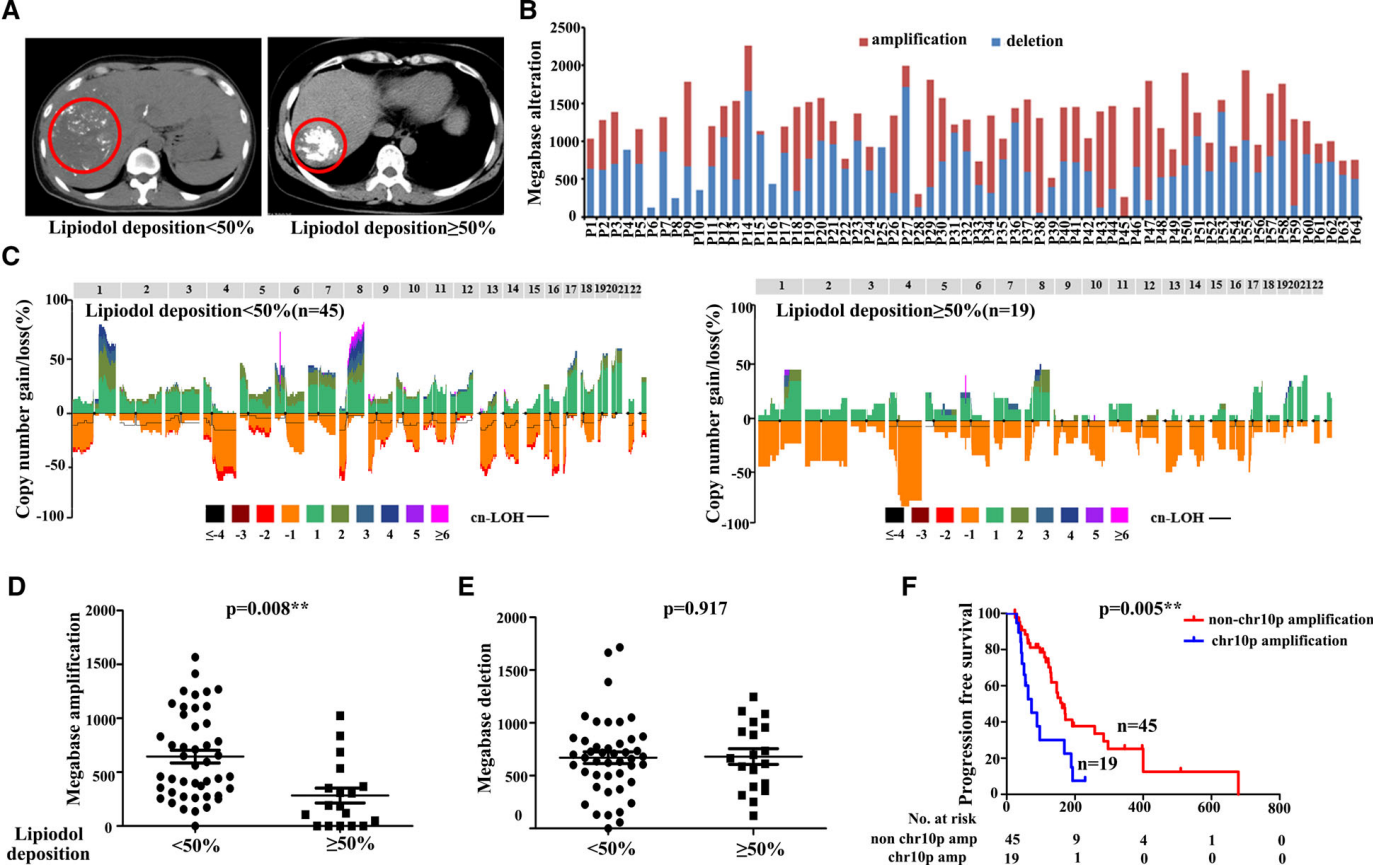
The correlation between lipiodol deposition and CNVs in cfDNA. (A) CT image showing lipiodol, left: lipiodol deposition <50%; right: lipiodol deposition ≥50%. (B) The size distribution of copy number amplification and deletion in each sample. (C) Copy number profile of lipiodol deposition <50% and lipiodol deposition ≥50% group. Patterns above the x‐axes represent amplification, whereas opposite contours indicate deletion and horizontal black lines indicate loss of heterozygosity. (D, E) The size of copy number amplification (D) and copy number deletions(E) was compared between lipiodol deposition < 50% and lipiodol deposition ≥ 50%, respectively. (F) Kaplan–Meier curves showing PFS for all enrolled patients stratified by chromosome 10p. Student's *t*‐test was used to compare the CNV alteration levels between the two lipiodol deposition groups. Statistical analysis for Kaplan–Meier plots used the log‐rank test. CNVs, copy number variants; CT, computed tomography.

Previous studies have demonstrated that lipiodol deposition in HCC tumour might be used as an imaging indicator to evaluate TACE therapeutic efficacy [21, 22, 23, 24]. By identifying the lipiodol deposition patterns on post‐TACE CT, we first calculated the proportion of lipiodol deposition in all enrolled patients and categorized them into two different groups with 50% as cut‐off according to previous reports [25, 26, 27] (Fig. 5A).

To explore whether pretreatment tumour genetic profiles could affect the lipiodol deposition process, we compared the copy number profile between these two groups of patients by calculating the total size of copy number amplification/loss regions in pre‐TACE plasma (Fig. 5B,C). Intriguingly, we found that the median size of copy number amplification in patients with lipiodol deposition < 50% is significantly higher than that in patients with lipiodol deposition ≥ 50% (645 versus 284 Mb, *P* = 0.008; Fig. 5D), while the size of copy number loss did not show any significant difference (*P* = 0.917; Fig. 5E). Furthermore, patients with high copy number amplification in chromosomes 1q, 3p, 6p, 8q, 10p, 12q, 18p and 18q had lower lipiodol deposition after TACE treatment (Table S7). Since lipiodol deposition play an important role in TACE’s tumour‐killing process, the copy number variations related to lipiodol deposition might affected patients' prognosis. As we expected, copy number variation, namely amplification of chromosome 10p in cfDNA, could lead to significantly shorter PFS (median survival 73 versus 163 days, *P* = 0.005; Figure 5F). The above results pointed out that CNVs could be a key factor determining lipiodol deposition in tumour tissue during TACE treatment, which in turn leads to the differences in patient's prognosis.

## 4 Discussion

TACE, as a locoregional palliative interventional therapy, is commonly applied on advanced HCC patients together with other treatment strategies (targeted therapy, immunotherapy, etc.). However, as a class of highly heterogeneous tumour, HCC patients often showed largely varied response to TACE treatment and there is an urgent need for developing an effective predictive biomarker to help guide clinical decision regarding to TACE treatment. Currently, the assessment of patients' response to TACE is mainly relying on the judgement of radiologists through imaging diagnosis, which is relatively subjective and highly affected by the experiences of the radiologists as well as numerous of other factors. For example, lipiodol deposition could cause beam hardening artefacts and increase blur on arterial phase, affecting the sensitivity of imaging approaches such as CT after TACE; coagulative haemorrhagic necrosis may cause high T1 signals in MRI, making it difficult for accurate evaluation. These limitations encouraged the development of nonimaging‐based approaches that could assist or supplement current strategy. In this study, we found that calculating cfDNA CNVs based on LD‐WGS could provide an accurate and cost‐efficiency strategy for evaluating the prognosis and monitoring the therapeutic efficacy of TACE treatment. TFx, which quantify the TFx in circulating system, could serve as a biomarker for patients' tumour burden and could real‐time monitor HCC progression, providing a potential alternative strategy aside from clinically applied biomarker AFP and mRECIST assessment system. We further discovered that the change in TFx between pre‐TACE and post‐TACE plasma samples could well predict patients' PFS.

Considering that cfDNA low‐depth sequencing is a relatively cheap and noninvasive approach to acquire tumour genetic information, this technology provided a quantifiable measurement for tumour burden and TACE response, which could better guide patients' personalized diagnosis and treatment. Blood sampling and low‐depth sequencing during patients' follow‐up could be clinically feasible for real‐time monitoring patients' status, benefiting corresponding treatment adjustment.

Chromosomal instability is a hallmark of tumour progression, and distinct copy number alteration patterns have been discovered in different cancers. For HCC, chromosomes 1q and 8q are frequently amplified and chromosomes 1p, 4q, 6q, 9p, 16p, 16q and 17p are frequently lost. The recurrent copy number amplification and loss have indicated that the copy number profile might affect patients' response to therapy. In our study, we found that patients with 16q amplification in cfDNA have significantly shorter PFS than that of patients without 16q amplification. These results suggested that chromosome 16q amplification in cfDNA may be related to poor TACE efficacy in HCC patients. Interestingly, a driver gene in 16q, namely NQO1, has been proved to be the major player between the 16q amplification and TACE response, with its amplification also significantly enriched in HCC patients with better clinical outcome. Previous reports have pointed out that NQO1 was significantly overexpressed in HCC tissue and involved in metabolic adaptation, which could affect glycolysis and glutaminolysis, resulting in uncontrolled rapid proliferation of cancer cells [28, 29]. However, we did not observed significant differences in blood glucose or glutamine (data no shown), which might be due to the involvement of other factors and limited sample size. Moreover, Shimokawa et al. [30] also indicated that reducing NQO1 activity in HCC tissue could intercept cancer cell anoikis resistance and reduce metastatic potential. Thus, our results further suggested that the amplification of this driver gene might lead to its overexpression, inducing tumour ischaemia/hypoxia resistance. Above observations have proved the importance of copy number profiling in the prediction of therapeutic efficiency, and further investigation into certain regions affected by recurrent copy number change might promote the discovery of potential biomarkers for better guiding treatment selection.

Lipiodol served as an important component for TACE drug delivery system, and lipiodol deposition in tumour can reduce drug washout and induce persistent effect. Numerous reports have pointed out the correlation between lipiodol deposition and tumour characteristics [21, 22]. As a molecular basis of tumour characteristics, the genetic profiles might also play an important part in the process of lipiodol deposition, which in turn still remained unexplored. In this study, we firstly analysed the potential factors influencing lipiodol deposition from the perspective of copy number variation and found that patients with chr1q, chr3p, chr6p, chr8q, chr10p, chr12q and chr18p amplification have worse lipiodol deposition, suggesting that the lipiodol deposition is indeed associated with HCC genetic profiles. This observation has laid the foundation for future investigation into the molecular mechanisms of lipiodol deposition, which could further help the development of clinical applicable strategies.

## 5 Conclusions

In conclusion, our study demonstrated that TFx evaluation and copy number profiling of cfDNA could serve as a robust strategy for evaluating TACE treatment outcomes in HCC patients. We also discovered how certain copy number amplification in cfDNA could affect lipiodol deposition and further have an impact on TACE response, which could be used to better optimize clinical decision to guide personalized TACE treatment. Future validation in large‐scale patient cohorts could improve our understanding of the correlations between cfDNA profiles and therapeutic efficacy of TACE.

## Conflict of interest

The authors declare no potential conflicts of interest.

### Peer Review

The peer review history for this article is available at https://publons.com/publon/10.1002/1878‐0261.13170.

## Author contributions

XQD, ZXC, XLL and JFL conceptualized and designed the study. XQD, XHH, HKC, YZ and LMQ involved in acquisition of data (organized patient enrolment, sample collection and clinical data curation). XQD, GC, ZLL, FP and LH analysed and interpreted the data (e.g. statistical analysis, biostatistics and computational analysis). XQD, GC, ZXC, XLL and JFL wrote, reviewed and/or revised the manuscript. ZXC, XLL and JFL supervised the study.

## Supporting information


**Table S1.** Associations between the levels of pre‐TACE TFx and clinicopathological characteristics of 64 advanced HCC patients.
**Table S2.** Multivariable analysis between the levels of pre‐TACE TFx and clinicopathological characteristics.
**Table S3.** Associations between the levels of post‐TACE TFx and clinicopathological characteristics of 64 advanced HCC patients.
**Table S4.** Multivariable analysis between the levels of pre‐TACE TFx and clinicopathological characteristics.
**Table S5.** The univariable analysis of TFx change and clinicopathological characteristics for PFS of 64 advanced HCC patients.
**Table S6.** All gains and losses of copy number alteration in pre‐TACE cfDNA between patients with TFx‐increase and patients with TFx‐decline or TFx‐stable.
**Table S7.** Associations between lipiodol deposition rate and clinicopathological characteristics of 64 advanced HCC patients.
**Fig. S1.** Comprehensive profiles of cfDNA and protein biomarker in HCC patients.
**Fig. S2.** The time‐course demonstration of TFx score (blue line), serum biomarker AFP level (orange line), tumor size (gray line) in long‐term follow‐up patients.

## Data Availability

The data that support the findings of this study are available from the corresponding author upon reasonable request.
