# Supplementary material for: Copy number profiling of circulating free DNA predicts transarterial chemoembolization response in advanced hepatocellular carcinoma
Source: Mol Oncol. 2022 Jan 10;16(10):1986–99. doi: 10.1002/1878-0261.13170 (PMC9120881; doi:10.1002/1878-0261.13170)

Table S1. Associations between the levels of pre-TACE TFx and clinicopathological characteristics of 64 advanced HCC patients.

|  |  | TFx＜0.1 | TFx ≥0.1 | p-value |
| --- | --- | --- | --- | --- |
| Sex | female | 5 | 2 | 0.168 |
|  | male | 25 | 32 |  |
| Age(years) | ＜55 | 11 | 16 | 0.401 |
|  | ≥55 | 19 | 18 |  |
| HBV-DNA(IU/ml) | ＜1000 | 18 | 11 | **0.027*** |
|  | ≥1000 | 12 | 23 |  |
| Tumor number | 1 | 10 | 8 | 0.384 |
|  | ＞1 | 20 | 26 |  |
| PVTT | Negative | 13 | 7 | **0.05*** |
|  | Positive | 17 | 27 |  |
| Tumor size(cm) | ＜5 | 11 | 0 | **＜0.001***** |
|  | ≥5 | 19 | 34 |  |
| BCLC | A-B | 8 | 3 | 0.059 |
|  | C | 22 | 31 |  |

Table S2. Multivariable analysis between the levels of pre-TACE TFx and clinicopathological characteristics

|  | multivariate analysis | |
| --- | --- | --- |
|  | HR (95% CI) | p-value |
| HBV-DNA(IU/ml) | 3.622(1.110-11.818) | **0.033*** |
| PVTT | 1.168(0.273-4.996) | 0.834 |
| Tumor size(cm) | **quasi-completely** | **quasi-completely** |

Table S3 Associations between the levels of post-TACE TFx and clinicopathological characteristics of 64 advanced HCC patients.

|  |  | TFx＜0.1 | TFx≥0.1 | p-value |
| --- | --- | --- | --- | --- |
| Sex | female | 6 | 1 | 0.206 |
|  | male | 35 | 22 |  |
| Age(years) | ＜55 | 15 | 12 | 0.226 |
|  | ≥55 | 26 | 11 |  |
| HBV-DNA(IU/ml) | ＜1000 | 22 | 7 | 0.073 |
|  | ≥1000 | 19 | 16 |  |
| Tumor number | 1 | 15 | 3 | **0.044*** |
|  | ＞1 | 26 | 20 |  |
| PVTT | Negative | 17 | 3 | **0.019*** |
|  | Positive | 24 | 20 |  |
| Tumor size(cm) | ＜5 | 15 | 1 | **0.004**** |
|  | ≥5 | 26 | 22 |  |
| BCLC | A-B | 8 | 3 | 0.510 |
|  | C | 33 | 20 |  |

Table S4. Multivariable analysis between the levels of pre-TACE TFx and clinicopathological characteristics

|  | multivariate analysis | |
| --- | --- | --- |
|  | HR (95% CI) | p-value |
| Tumor number | 3.609(00.843-15.452) | 0.084 |
| PVTT | 2.886(0.654-12.736) | 0.162 |
| Tumor size(cm) | 8.808(1.005-77.178) | **0.049*** |

Table S5. The univariable analysis of TFx change and clinicopathological characteristics for PFS of 64 advanced HCC patients.

|  | Univariate | | |
| --- | --- | --- | --- |
|  | HR (95% CI) | | P value |
| Sex, female *vs.* male | | 1.916 (0.589-6.231) | 0.280 |
| Age(years), <55 *vs.* ≥55 | | 0.647 (0.351-1.195) | 0.164 |
| HBV-DNA(IU/ml), <1000 *vs.* ≥1000 | | 1.000(1.000-1.000) | 0.751 |
| Tumor number. =1 *vs.*＞1 | | 1.754(0.823-3.739) | 0.146 |
| PVTT, Absent *vs.* Present | | 1.401(0.723-2.716) | 0.318 |
| BCLC，A-B *vs.* C | | 2.100(0.876-5.032) | 0.096 |
| Tumor size(cm),＜5 *vs.* ≥5 | | 1.060(0.989-1.136） | 0.098 |
| TFx change, TFx decline/stable *vs.* TFx increase | | 1.809 (1.143-2.863) | **0.011*** |

Table S6. All gains and losses of copy number alteration in pre-TACE cfDNA between patients with TFx-increase and patients with TFx-decline or TFx-stable.

|  | TFx decline/stable | | TFx increase | |  |  | TFx decline/stable | | TFx increase | |  |
| --- | --- | --- | --- | --- | --- | --- | --- | --- | --- | --- | --- |
|  | amp | non-amp | amp | non-amp | p value |  | del | non-  del | del | non-  del | p value |
| chr1p | 14 | 43 | 4 | 3 | 0.173 |  | 25 | 32 | 2 | 5 | 0.713 |
| chr1q | 41 | 16 | 5 | 2 | 1.000 |  | 9 | 48 | 0 | 7 | 0.577 |
| chr2p | 15 | 42 | 1 | 6 | 0.817 |  | 11 | 46 | 2 | 5 | 0.938 |
| chr2q | 14 | 43 | 3 | 4 | 0.561 |  | 18 | 39 | 3 | 4 | 0.862 |
| chr3p | 16 | 41 | 4 | 3 | 0.257 |  | 13 | 44 | 1 | 6 | 0.976 |
| chr3q | 14 | 43 | 2 | 5 | 1.000 |  | 8 | 49 | 0 | 7 | 0.650 |
| chr4p | 18 | 39 | 5 | 2 | 0.098 |  | 20 | 37 | 3 | 4 | 1.000 |
| chr4q | 8 | 49 | 1 | 6 | 1.000 |  | 40 | 17 | 5 | 2 | 1.000 |
| chr5p | 23 | 34 | 3 | 4 | 1.000 |  | 2 | 55 | 1 | 6 | 0.745 |
| chr5q | 15 | 42 | 3 | 4 | 0.636 |  | 13 | 44 | 2 | 5 | 1.000 |
| chr6p | 39 | 18 | 4 | 3 | 0.862 |  | 9 | 48 | 0 | 7 | 0.577 |
| chr6q | 19 | 38 | 3 | 4 | 0.937 |  | 23 | 34 | 2 | 5 | 0.847 |
| chr7p | 21 | 36 | 4 | 3 | 0.530 |  | 9 | 48 | 0 | 7 | 0.577 |
| chr7q | 21 | 36 | 4 | 3 | 0.530 |  | 12 | 45 | 0 | 7 | 0.404 |
| chr8p | 14 | 43 | 4 | 3 | 0.173 |  | 31 | 26 | 4 | 3 | 1.000 |
| chr8q | 42 | 15 | 7 | 0 | 0.281 |  | 9 | 48 | 1 | 6 | 1.000 |
| chr9p | 12 | 45 | 1 | 6 | 1.000 |  | 29 | 28 | 3 | 4 | 1.000 |
| chr9q | 9 | 48 | 2 | 5 | 0.753 |  | 18 | 39 | 2 | 5 | 1.000 |
| chr10p | 17 | 40 | 2 | 5 | 1.000 |  | 9 | 48 | 1 | 6 | 1.000 |
| chr10q | 13 | 44 | 2 | 5 | 1.000 |  | 23 | 34 | 1 | 6 | 0.352 |
| chr11p | 14 | 43 | 2 | 5 | 1.000 |  | 11 | 46 | 0 | 7 | 0.455 |
| chr11q | 20 | 37 | 3 | 4 | 1.000 |  | 15 | 42 | 0 | 7 | 0.281 |
| chr12p | 9 | 48 | 1 | 6 | 1.000 |  | 17 | 40 | 2 | 5 | 1.000 |
| chr12q | 18 | 39 | 3 | 4 | 0.862 |  | 10 | 47 | 1 | 6 | 1.000 |
| chr13p | 0 | 57 | 0 | 7 | NA |  | 0 | 57 | 0 | 7 | NA |
| chr13q | 14 | 43 | 1 | 6 | 0.894 |  | 35 | 22 | 2 | 5 | 0.210 |
| chr14p | 0 | 57 | 0 | 7 | NA |  | 0 | 57 | 0 | 7 | NA |
| chr14q | 15 | 42 | 2 | 5 | 1.000 |  | 27 | 30 | 3 | 4 | 1.000 |
| chr15p | 0 | 57 | 0 | 7 | NA |  | 0 | 57 | 0 | 7 | NA |
| chr15q | 11 | 46 | 0 | 7 | 0.455 |  | 19 | 38 | 1 | 6 | 0.552 |
| chr16p | 10 | 47 | 3 | 4 | 0.283 |  | 21 | 36 | 3 | 4 | 1.000 |
| chr16q | 5 | 52 | 3 | 4 | **0.049*** |  | 31 | 26 | 3 | 4 | 0.861 |
| chr17p | 10 | 47 | 3 | 4 | 0.283 |  | 32 | 25 | 2 | 5 | 0.328 |
| chr17q | 28 | 29 | 5 | 2 | 0.475 |  | 8 | 49 | 0 | 7 | 0.650 |
| chr18p | 16 | 41 | 3 | 4 | 0.712 |  | 7 | 50 | 1 | 6 | 1.000 |
| chr18q | 14 | 43 | 3 | 4 | 0.561 |  | 14 | 43 | 1 | 6 | 0.894 |
| chr19p | 22 | 35 | 5 | 2 | 0.210 |  | 16 | 41 | 0 | 7 | 0.248 |
| chr19q | 29 | 28 | 5 | 2 | 0.531 |  | 5 | 52 | 0 | 7 | 0.944 |
| chr20p | 29 | 28 | 5 | 2 | 0.531 |  | 2 | 55 | 1 | 6 | 0.745 |
| chr20q | 30 | 27 | 5 | 2 | 0.589 |  | 0 | 57 | 1 | 6 | 0.207 |
| chr21p | 0 | 57 | 0 | 7 | NA |  | 0 | 57 | 0 | 7 | NA |
| chr21q | 6 | 51 | 2 | 5 | 0.449 |  | 20 | 37 | 1 | 6 | 0.497 |
| chr22p | 0 | 57 | 0 | 7 | NA |  | 0 | 57 | 0 | 7 | NA |
| chr22q | 17 | 40 | 3 | 4 | 0.787 |  | 12 | 45 | 2 | 5 | 1.000 |

amp: amplification; del: deletion

Table S7. Associations between lipiodol deposition rate and clinicopathological characteristics of 64 advanced HCC patients.

|  | lipiodol deposition＜50% | | lipiodol deposition≥50% | |  |
| --- | --- | --- | --- | --- | --- |
|  | amp | non-amp | amp | non-amp | p value |
| chr1p | 13 | 32 | 5 | 14 | 1.000 |
| chr1q | 37 | 8 | 9 | 10 | **0.011*** |
| chr2p | 14 | 31 | 2 | 17 | 0.155 |
| chr2q | 13 | 32 | 4 | 15 | 0.735 |
| chr3p | 18 | 27 | 2 | 17 | **0.042*** |
| chr3q | 12 | 33 | 4 | 15 | 0.874 |
| chr4p | 17 | 28 | 6 | 13 | 0.852 |
| chr4q | 8 | 37 | 1 | 18 | 0.356 |
| chr5p | 21 | 24 | 5 | 14 | 0.216 |
| chr5q | 15 | 30 | 3 | 16 | 0.262 |
| chr6p | 35 | 10 | 8 | 11 | **0.013*** |
| chr6q | 18 | 27 | 4 | 15 | 0.242 |
| chr7p | 20 | 25 | 5 | 14 | 0.281 |
| chr7q | 21 | 24 | 4 | 15 | 0.101 |
| chr8p | 12 | 33 | 6 | 13 | 0.924 |
| chr8q | 39 | 6 | 10 | 9 | **0.009**** |
| chr9p | 11 | 34 | 2 | 17 | 0.355 |
| chr9q | 10 | 35 | 1 | 18 | 0.200 |
| chr10p | 18 | 27 | 1 | 18 | **0.013*** |
| chr10q | 14 | 31 | 1 | 18 | 0.056 |
| chr11p | 12 | 33 | 4 | 15 | 0.874 |
| chr11q | 20 | 25 | 3 | 16 | 0.058 |
| chr12p | 10 | 35 | 0 | 19 | 0.063 |
| chr12q | 19 | 26 | 2 | 17 | **0.030*** |
| chr13p | 0 | 45 | 0 | 19 | NA |
| chr13q | 13 | 32 | 2 | 17 | 0.207 |
| chr14p | 0 | 45 | 0 | 19 | NA |
| chr14q | 15 | 30 | 2 | 17 | 0.115 |
| chr15p | 0 | 45 | 0 | 19 | NA |
| chr15q | 8 | 37 | 3 | 16 | 1.000 |
| chr16p | 12 | 33 | 1 | 18 | 0.109 |
| chr16q | 7 | 38 | 1 | 18 | 0.469 |
| chr17p | 11 | 34 | 2 | 17 | 0.355 |
| chr17q | 26 | 19 | 7 | 12 | 0.209 |
| chr18p | 18 | 27 | 1 | 18 | **0.013*** |
| chr18q | 16 | 29 | 1 | 18 | **0.028*** |
| chr19p | 22 | 23 | 5 | 14 | 0.163 |
| chr19q | 26 | 19 | 8 | 11 | 0.382 |
| chr20p | 27 | 18 | 7 | 12 | 0.155 |
| chr20q | 27 | 18 | 8 | 11 | 0.299 |
| chr21p | 0 | 45 | 0 | 19 | NA |
| chr21q | 7 | 38 | 1 | 18 | 0.469 |
| chr22p | 0 | 45 | 0 | 19 | NA |
| chr22q | 15 | 30 | 5 | 14 | 0.796 |

amp: amplification; del: deletion

Fig. S1. Comprehensive profiles of cfDNA and protein biomarker in HCC patients. A. TFx levels in healthy volunteers, LC patients and HCC patients. B. The performance for TFx by ROC curve analysis. C. The distribution of serum biomarker AFP level (log2AFP) before TACE in each sample. D. Copy number profile of enrolled 64 HCC patients. E. The distribution of AFP level after TACE in each sample.

Fig. S2. The time-course demonstration of TFx score (blue line), serum biomarker AFP level (orange line), tumor size (gray line) in long-term follow-up patients. Black dotted line indicated tumor progression; red dotted line indicated TFx score could earlier predict tumor progression.

Fig. S1


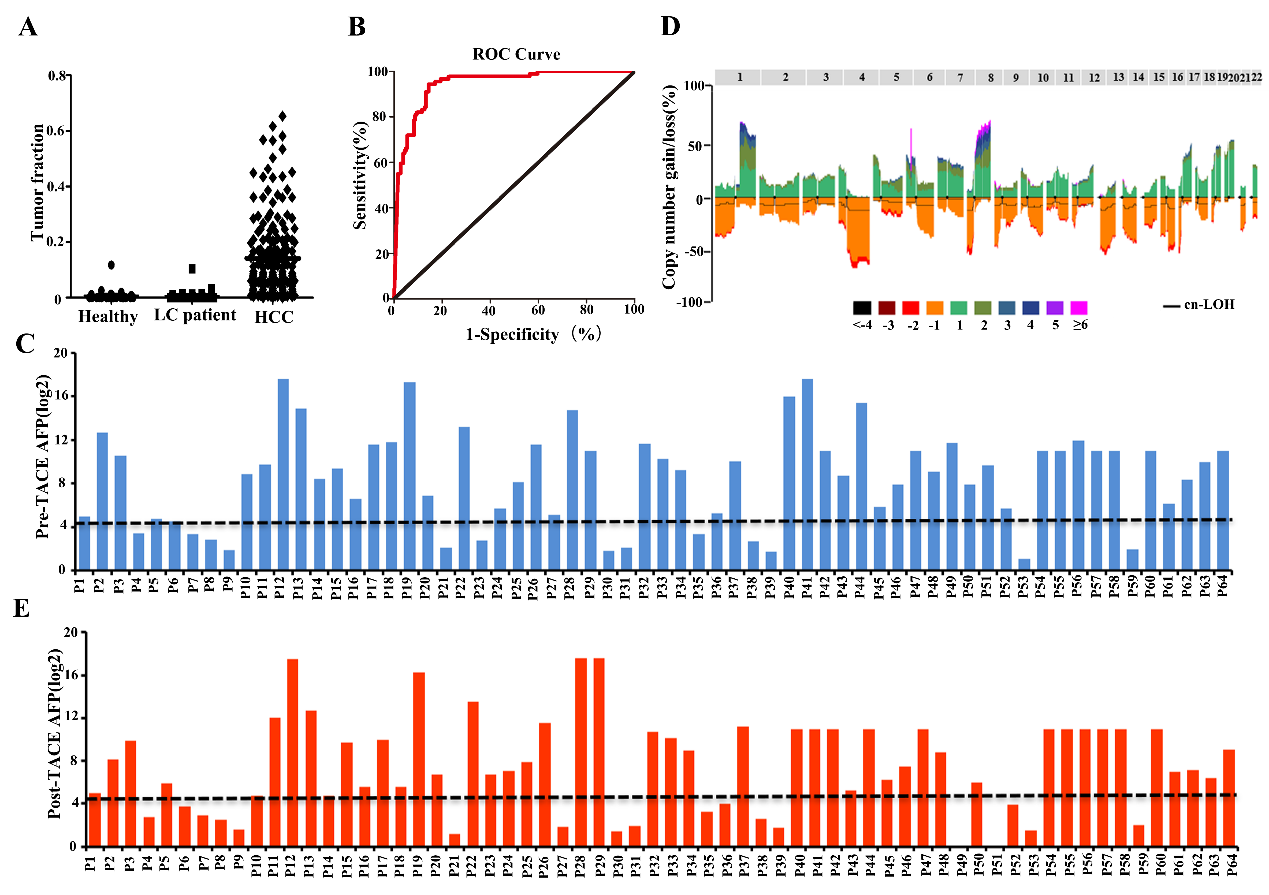


Fig. S2


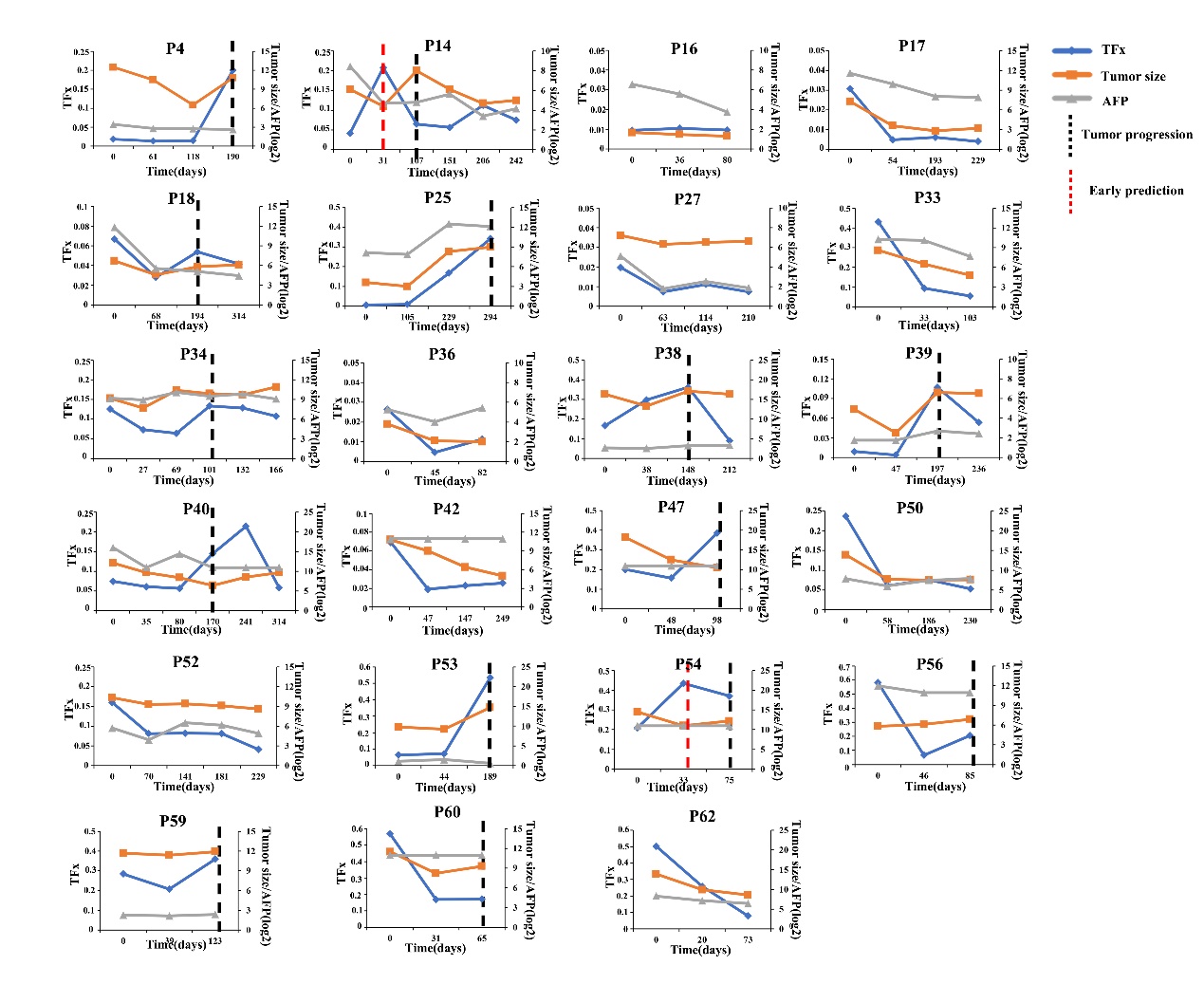

Supplement: Supplementary file 1 — Table S1. Associations between the levels of pre‐TACE TFx and clinicopathological characteristics of 64 advanced HCC patients. Table S2. Multivariable analysis between the levels of pre‐TACE TFx and clinicopathological characteristics. Table S3. Associations between the levels of post‐TACE TFx and clinicopathological characteristics of 64 advanced HCC patients. Table S4. Multivariable analysis between the levels of pre‐TACE TFx and clinicopathological characteristics. Table S5. The univariable analysis of TFx change and clinicopathological characteristics for PFS of 64 advanced HCC patients. Table S6. All gains and losses of copy number alteration in pre‐TACE cfDNA between patients with TFx‐increase and patients with TFx‐decline or TFx‐stable. Table S7. Associations between lipiodol deposition rate and clinicopathological characteristics of 64 advanced HCC patients. Fig. S1. Comprehensive profiles of cfDNA and protein biomarker in HCC patients. Fig. S2. The time‐course demonstration of TFx score (blue line), serum biomarker AFP level (orange line), tumor size (gray line) in long‐term follow‐up patients. [file MOL2-16-1986-s001.docx]
